# Neuroprotective Effect of *Brassica oleracea* Sprouts Crude Juice in a Cellular Model of Alzheimer's Disease

**DOI:** 10.1155/2015/781938

**Published:** 2015-06-09

**Authors:** Alessandra Masci, Roberto Mattioli, Paolo Costantino, Simona Baima, Giorgio Morelli, Pasqualina Punzi, Cesare Giordano, Alessandro Pinto, Lorenzo Maria Donini, Maria d'Erme, Luciana Mosca

**Affiliations:** ^1^Department of Experimental Medicine-Medical Physiopathology, Food Science and Endocrinology Section, Sapienza University, Rome, Italy; ^2^Department of Biology and Biotechnology, Sapienza University, Rome, Italy; ^3^Food and Nutrition Research Centre, Agricultural Research Council (CRA), Rome, Italy; ^4^Department of Chemistry, Sapienza University, Rome, Italy; ^5^Institute of Biology, Molecular Medicine and Nanobiotechnologies, National Research Council (CNR), Rome, Italy; ^6^Department of Biochemical Sciences, Sapienza University, Rome, Italy

## Abstract

*β*-Amyloid peptide (A*β*) aberrant production and aggregation are major factors implicated in the pathogenesis of Alzheimer's disease (AD), causing neuronal death *via* oxidative stress. Several studies have highlighted the importance of polyphenolic antioxidant compounds in the treatment of AD, but complex food matrices, characterized by a different relative content of these phytochemicals, have been neglected. In the present study, we analyzed the protective effect on SH-SY5Y cells treated with the fragment A*β*_25–35_ by two crude juices of broccoli sprouts containing different amounts of phenolic compounds as a result of different growth conditions. Both juices protected against A*β*-induced cytotoxicity and apoptotic cell death as evidenced by cell viability, nuclear chromatin condensation, and apoptotic body formation measurements. These effects were mediated by the modulation of the mitochondrial function and of the *HSP70* gene transcription and expression. Furthermore, the juices upregulated the intracellular glutathione content and mRNA levels or activity of antioxidant enzymes such as heme oxygenase-1, thioredoxin, thioredoxin reductase, and NAD(P)H:quinone oxidoreductase 1 *via* activation of NF-E2-related factor 2 (Nrf2). Although the effects of the two juices were similar, the juice enriched in phenolic compounds showed a greater efficacy in inducing the activation of the Nrf2 signalling pathway.

## 1. Introduction

The incidence of neurodegenerative diseases, such as Alzheimer's disease (AD), increases as a function of age. Their aetiology may partially involve lifestyle determinants such as hypercaloric diets poor in fruit and vegetables and sedentary lifestyle, which may lead to obesity, decreased insulin sensitivity, and metabolic syndrome; all of these are characterized by a low-grade inflammation [1]. Due to the steady increase in life expectancy, the prevalence of neurodegenerative diseases is calculated to double within the next 30 years implying that unless ways are found to reduce age-related cognitive decline, healthcare costs will rise exponentially, with a deep social impact and serious implications in terms of economic burden [2]. Hence, therapeutic tools are actively sought which can counteract the neurodegenerative processes and many drugs have been developed and tested in various models and in humans. However, in spite of the promising effects observed in preclinical studies, in most cases clinical trials failed to show therapeutic effectiveness or to reverse the disease course. One possible alternative strategy would be to operate on lifestyle determinants, in particular on physical activity level and nutrition.

Polyphenolic compounds contained in vegetables, fruits, nuts, and spices exhibit remarkable antioxidant and anti-inflammatory activities which may exert an important role in reducing age-related oxidative stress and inflammation thus hampering the neurodegenerative processes [3, 4]. In this perspective, functional foods and nutraceuticals enriched in polyphenols may represent a novel therapeutic approach in view of their ability to exert anti-inflammatory and antioxidant properties. A large amount of evidence has accumulated over the past few years which strongly implicates free radical-induced oxidative damage in the pathogenesis of several neurodegenerative diseases. The brain is particularly susceptible to oxidative stress due to its extremely high consumption of oxygen and glucose, high content in polyunsaturated fatty acids, and paucity of antioxidant defense systems [5].* Postmortem* studies on brain specimens collected from individuals affected by AD revealed an extensive oxidative stress compared to healthy controls,* that is*, increased levels of oxidative markers of lipid, protein, and DNA damage. Therefore, a role for the antioxidant polyphenols in the prevention and/or treatment of AD has been hypothesized [6].

Brassica vegetables are among the foods of increasing interest in nutrition science as a consequence of the beneficial effects of their different phytochemicals on human health [7]. The genus* Brassica* (family* Brassicaceae*, also known as* Cruciferae*) includes a high number of vegetables comprising amongst others broccoli, cauliflower, Brussels sprouts, kohlrabi, cabbage, and mustard. Several epidemiologic studies highlight that a regular intake of Brassica vegetables is associated with a reduced incidence of cancer, and further beneficial effects on health were demonstrated in cardiovascular diseases and in metabolic disorders such as diabetes [8, 9]. Indeed, the edible species within the* Brassicaceae* are a good source of many health promoting compounds including glucosinolates and their by-products, especially isothiocyanates (ITCs) such as sulforaphane (SFN), phenolics, vitamins, carotenoids, proteins, sugars, chlorophyll, minerals, fatty acids, and amino acids [10, 11]. Among phytochemicals contained in broccoli, the most studied are glucosinolates and related compounds together with phenolics [7]. SFN and phenolics (in particular flavonoids) exhibit anticarcinogenic, anti-inflammatory, antioxidant, chemopreventive, and cytoprotective properties [12–14]. Recently, it has been reported that SFN and flavonoids can penetrate the blood brain barrier and exert neuroprotective effects in animal models of neurological disorders [15–18]. In addition, SFN and polyphenols have been reported to attenuate microglia-induced inflammation in hippocampus of LPS-treated mice and microglia cell lines [19–21].

Although to date several evidences support the neuroprotective role of individual, purified broccoli phytochemicals, few studies have taken into account the potential protective effects of complex matrices of bioactive molecules contained in broccoli. Currently, nutrition research tends to emphasize the importance of assessing the interactions among the various phytochemicals in food or in their crude derivatives, with regard to their effects on the pathophysiology of the human body. In this work broccoli sprouts of* Brassica oleracea* L. var.* botrytis *subvar.* cymosa* were grown in two conditions,* that is*, in the dark or exposed to white light in the presence of sucrose, in order to obtain a clear difference in terms of the phenolics/anthocyanins content between the differently grown sprouts. Juices obtained by cold pressing of the sprouts were then assayed for their antioxidant capacity and for their ability to act as possible neuroprotective agents in a cellular model of AD,* that is*, SH-SY5Y human neuroblastoma cells treated with the 25–35 fragment of the *β*-amyloid peptide (A*β*_25–35_), one of the most toxic fragments of the full length A*β*_1–42_. In particular, we investigated whether broccoli sprouts juices can attenuate A*β*_25–35_-induced cytotoxicity and cell death by stimulating the antioxidant defence capacity* via* activation of NF-E2-related factor 2 (Nrf2) and the subsequent expression of antioxidant and phase II detoxification enzymes which play key roles in counteracting oxidative damages.

## 2. Materials and Methods

### 2.1. Materials

Unless otherwise stated, reagents were purchased from Sigma Aldrich (St. Louis, MO, USA). A*β*_25–35_ was synthesized by conventional solid phase chemistry [22]. Other reagents were Trizol reagent from Invitrogen (Carlsberg, CA, USA); QuantiTect Reverse Transcription kit from QIAGEN (Hilden, Germany); primary antibody rabbit monoclonal anti-Nrf2 and secondary antibody goat polyclonal anti-rabbit IgG (Alexa Fluor 488) from Abcam (Cambridge, UK).

### 2.2. Plant Growth and Juice Preparation

Broccoli sprouts (*Brassica oleracea* L. var.* botrytis* subvar.* cymosa*) were grown essentially as described by Maldini et al. [23]. After sterilization the seeds were transferred into the Vitaseed (SUBA & UNICO, Longiano, FC, Italy) germinator filled with distilled water. Sprouts were grown for 5 days at 21°C and 70% humidity in a dedicated climatic chamber (Weiss Gallenkamp, Loughborough, UK) in the dark (sprouts of type A) or with 16 h of illumination and 8 h of darkness (sprouts of type B), respectively. After the first 3 days of growth the sprouts of type B were treated for 48 h with a solution of 176 mM sucrose, freshly prepared with sterile distilled water. At the end of the 5 days of growth, plantlets were weighed and cold-pressed with Angel 8500S Luxury juicer (Living Juice Ltd., Lecco, LC, Italy) for the production of juice. The juices obtained were centrifuged at 4,000 g for 30 min at 4°C. The supernatant was immediately frozen in liquid nitrogen and stored at −80°C until use. Before treating the cells, the juices were diluted in the culture medium and filtered on a 0.2 *μ*m sterile cellulose acetate membrane.

### 2.3. Determination of Total Phenols, Flavonoids, Anthocyanins, and Sulforaphane

#### 2.3.1. Total Phenols

Total phenols were determined by the Folin-Ciocalteu assay as described by Singleton and Rossi Jr. [24]. Briefly, the reaction solution was prepared by mixing 10 *μ*L of blank, standard, or sample with 790 *μ*L of distilled water. After addition of 50 *μ*L of Folin-Ciocalteu reagent the reaction mixture was incubated for 3 min at room temperature (RT) and then 150 *μ*L of a 20% (w/v) Na_2_CO_3_ aqueous solution was added. After 2 h of incubation, the absorbance at 760 nm was measured on a Hitachi U2000 spectrophotometer (Hitachi, Tokyo, Japan). The results were expressed as mg of gallic acid equivalents (GAE) per mL of juice.

#### 2.3.2. Total Flavonoids

The total flavonoid content of the juices was determined using the aluminium trichloride assay as described by Dewanto et al. [25]. The assay was carried out in a 96-well plate and in each well the following solutions were added: 20 *μ*L of rutin hydrate standard (half serial dilutions of 1 mg/mL) or 20 *μ*L of a dilution of juices or blank, 20 *μ*L of sodium nitrate solution (3%, w/v), 20 *μ*L of aluminum trichloride solution (1%, w/v), and 100 *μ*L of sodium hydroxide solution (0.5 M). Absorbance was measured at 450 nm (Appliskan microplate reader, Thermo Scientific, Vantaa, Finland). Results were expressed as mg of rutin equivalents (RE) per mL of juice.

#### 2.3.3. Total Anthocyanins

Total anthocyanins quantification was performed by the pH-differential method as described by Giusti and Wrolstad [26]. The juice was diluted in a pH 1.0 solution (0.1 M HCl, 25 mM KCl) and in a pH 4.5 solution (0.4 M CH_3_COONa). The absorbance of the mixtures was then measured at 535 and 700 nm against distilled water. The value (Abs_535_–Abs_700_)_pH1.0_–(Abs_535_–Abs_700_)_pH4.5_ corresponds to the absorbance due to the anthocyanins. Calculation of the anthocyanins concentration was based on a cyanidin-3-*O*-glucoside (Cy-3G) molar extinction coefficient of 25,965 M^−1^  × cm^−1^ and a molecular mass of 449.2 g × mol^−1^. Results were expressed as *μ*g cyanidin-3-*O*-glucoside equivalents (CGE) per mL of juice.

#### 2.3.4. Sulforaphane

SFN determination was performed using an HPLC system (Perkin-Elmer, USA) interfaced to an Applied Biosystems (Foster City, CA, USA) API 3200 Q-Trap spectrometer. Quantitative on-line HPLC-ESI-MS/MS analyses were performed using mass spectrometer in Multiple Reaction Monitoring (MRM) mode. The API 3200 ES source was tuned by infusing a standard solution of SFN (1 *μ*g/mL in methanol 50%, v/v) into the source at a flow rate of 10 *μ*L/min. The optimized parameters were declustering potential 45 eV, entrance potential 5 eV; fragmentation reactions selected for SFN were 178 → 114 (CE = 18; CXP = 4; CEP = 14). The source temperature was held at 400°C and the voltage applied was −4500. The dwell time was 120 ms. Samples juices were opportunely diluted in H_2_O with 0.1% (v/v) formic acid, filtered, injected (10 *μ*L) into a Luna C18 column (Phenomenex, USA) (5 *μ*m, 150 × 2.1 mm), and eluted at flow rate of 0.3 mL/min. Mobile phase A was H_2_O containing 0.1% (v/v) formic acid while mobile phase B was acetonitrile containing 0.1% (v/v) formic acid. Elution gradient was from 100% A to 20 : 80 (A : B) in 20 min and from 20 : 80 (A : B) to 0 : 100 (A : B) in 1 min. The column was kept at 25°C, using a Peltier Column Oven Series 200 (Perkin Elmer). Data acquisition and processing were performed using Analyst software 1.5.1. SFN concentration was calculated over an external standard curve of SFN.

### 2.4. Antioxidant Activity

#### 2.4.1. NBT Assay

Broccoli sprouts juices effect as superoxide anion scavenger was assayed by the inhibition of nitroblue tetrazolium chloride (NBT) reduction by *β*-nicotinamide adenine dinucleotide reduced form (*β*NADH) in the presence of phenazine methosulfate (PM) as described by Yuting et al. [27]. Reaction mixtures contained 73 *μ*M *β*NADH, 15 *μ*M PM, 50 *μ*M NBT, and 0–20 *μ*L/mL of A or B juice in 1 mL of 0.02 M Tris-HCl buffer, pH 8.0. Absorbance variations (ΔAbs/min) were determined at 560 nm, by measuring the initial rate of superoxide anion-induced NBT reduction. The percentage inhibition of ΔAbs_560 nm_/min after 15 sec at 25°C was calculated and plotted as a function of concentration of antioxidants. Stock solutions (1 mM) were freshly prepared every day dissolving PM in ethanol, NBT in water, and *β*NADH in 0.05 M phosphate buffer, pH 7.4.

#### 2.4.2. ABTS Assay

The 2,2′-azinobis(3-ethylbenzothiazoline-6-sulfonic acid) diammonium salt (ABTS) was dissolved in water to a 7 mM final concentration. ABTS radical cation (ABTS^•+^) was produced by reacting ABTS stock solution with 2.45 mM potassium persulfate for 16 h in the dark at RT [28]. ABTS^•+^ was diluted with ethanol, to obtain an absorbance of 0.70 (±0.02) at 734 nm. 10 *μ*L of diluted juices in ethanol (0–10 *μ*L/mL final concentration) was added to 1 mL of the working ABTS^•+^ solution. The percentage inhibition of absorbance at 734 nm after 6 min at RT was calculated and plotted as a function of antioxidant concentration.

#### 2.4.3. DPPH Assay

An aliquot of 10 *μ*L of diluted juices in ethanol (0–10 *μ*L/mL final concentration) was added to 1 mL of 30 *μ*M ethanolic solution of stable nitrogen centered free radical 2,2-diphenyl-1-picrylhydrazyl (DPPH^•^) and the absorbance was monitored spectrophotometrically at 517 nm after 15 min at RT. Radical DPPH^•^ scavenging capacity was estimated from the difference in absorbance with or without antioxidants and expressed as percent DPPH^•^ disappearance as a function of the sample concentration.

#### 2.4.4. Deoxyribose Assay

The Fe(II)-H_2_O_2_-induced degradation of 2-deoxy-d-ribose in the presence of EDTA was assayed as previously described by Halliwell et al. [29]. Briefly, 1 mL reaction mixture contained 2.8 mM 2-deoxy-d-ribose, 0.1 M phosphate buffer (pH 7.4), 0–20 *μ*L juice A or juice B, 50 *μ*M ammonium ferrous sulphate ((NH_4_)_2_Fe(SO_4_)_2_), 0.1 mM EDTA, and 1 mM hydrogen peroxide (H_2_O_2_). Fresh solution of Fe(II) was prepared in deaerated water immediately before each experiment and used to start the reactions which were carried out at 37°C for 30 min. The extent of 2-deoxy-d-ribose degradation was monitored by the formation of malondialdehyde determined by the addition of 1 mL of 1% (w/v) 2-thiobarbituric acid in 50 mM sodium hydroxide and 1 mL of 2.8% (w/v) trichloroacetic acid. After heating at 80°C for 20 min, the reaction solutions were cooled and the absorbance was read at 532 nm against appropriate blanks. The scavenger activity towards OH^•^ radical was expressed as percentage inhibition of 2-deoxy-d-ribose degradation in the presence of antioxidants compared to the control.

#### 2.4.5. ORAC Assay

For oxygen radical absorbance capacity (ORAC) assay, the methods of Cao and Prior [30] were used. The assay was carried out in black-walled 96-well plates and each well contained a final volume of 200 *μ*L. Broccoli sprouts juices were diluted with 75 mM phosphate buffer (pH 7.0) to a final concentration of 0.5 *μ*L/mL. The (±)6-hydroxy-2,5,7,8-tetramethylchroman-2-carboxylic acid (Trolox) was used as a standard to a final concentration of 5 *μ*M. The reaction solution also contained 3.4 *μ*g/mL of R-phycoerythrin, and the plate was incubated at 37°C for 15 min. Then 2,2′-azobis(2-methylpropionamidine) dihydrochloride was added to each well to a final concentration of 16 mM, and fluorescence intensity was estimated every 5 min for a total of 120 min using an excitation filter of 492/10 nm and an emission filter of 570/10 nm. Results were calculated on the basis of the differences in area under the curve between the control and the sample.

### 2.5. Preparation of Aggregated A*β*_25–35_

A*β*_25–35_ was dissolved in sterile phosphate buffered saline, pH 7.4 (PBS) at a concentration of 1 mM, and incubated in a Sonicator Bath at RT for 15–30 min to induce aggregation. After aggregation, the solution was stored at −20°C until use. Immediately before treating the cells, stock solution was diluted to 25 *μ*M final concentration in culture medium.

### 2.6. Cell Culture

Human neuroblastoma SH-SY5Y cell line was obtained from the ICLC (Genova, Italy). Cells were grown in DMEM/F-12 medium containing 10% fetal bovine serum (Gibco BRL. Life Technologies Inc., Grand Island, NY, USA) and 2 mM l-glutamine at 37°C in a humidified atmosphere with 5% CO_2_. Cells were plated at an appropriate density according to each experimental setting and treated with 25 *μ*M aggregated A*β*_25–35_ in the presence or in the absence of 10 *μ*L/mL of juice A or juice B. Appropriate controls in which the cells were treated only with juice A or juice B were run in parallel.

### 2.7. Cell Viability Assay

Cell viability was determined by using thiazolyl blue tetrazolium bromide (MTT) dye reduction assay. Briefly, cells were seeded in 96-well plates at a density of 15,000 cells/well. After treatment, 20 *μ*L of a 5 mg/mL solution of MTT in PBS was added to the culture medium and cells were incubated at 37°C for 2 h. The supernatants were then aspirated off and formazan crystals were dissolved with 100 *μ*L/well of dimethyl sulfoxide. The optical density of each well was determined at 570 nm with a reference at 690 nm using a microplate reader.

### 2.8. Assessment of Apoptosis by Nuclear Staining

Apoptosis was analyzed by using the fluorescent dye Hoechst 33258 [31]. Briefly, cells were seeded on 18 × 18 mm glass coverslips in 6-well plates at a density of 200,000 cells/well. After treatment, the cells were washed in PBS and fixed in 4% (w/v) formaldehyde for 20 min. The coverslips were permeabilized for 5 min in 0.5% (v/v) Triton X-100 and then stained with 0.1 *μ*g/mL Hoechst 33258. Coverslips were mounted with 50% (v/v) glycerol in PBS and examined by fluorescence microscope (DM IL LED model, Leica Microsystems CMS Gmb H, Wetzlar, Germany) equipped with an 355 nm excitation filter, an 465 nm emission filter, and a digital camera (Leica DCF450 C). For each slide 5 images were collected at random position by means of the Leica Application Suite Advanced Fluorescence (LAS AF) software. Apoptotic cells were recognized by their characteristic nuclei condensation, fragmentation, and bright staining.

### 2.9. Flow Cytometric Detection of Apoptotic Cells

Apoptotic cells can be recognized after staining with a DNA-specific fluorochrome by flow cytometry as having less DNA than G1-cells. Cells were plated in 6-well plates at a density of 450,000 cells/well. After treatment cells were collected by centrifugation, suspended in ice-cold 70% (v/v) ethanol in PBS, and fixed at 4°C for 48 h, then the cells were washed and suspended in 600 *μ*L of DNA staining reagent containing 180 *μ*g/mL ribonuclease A and 50 *μ*g/mL propidium iodide (PI). Red fluorescence (DNA) was detected by a flow cytometer (BD Accuri C6, BD Bioscences, Erembodegem, Belgium) equipped with a 488 nm excitation laser and a 585/40 nm band-pass filter (FL2 channel). 30,000 events were collected for each sample and the percentage of apoptotic cells accumulating in the sub-G1 peak was calculated.

### 2.10. Measurement of Mitochondrial Membrane Potential

The mitochondrial membrane potential (ΔΨm) value was determined by measuring the change in red/green fluorescence emission of the mitochondrial probe 5,5′,6,6′-tetrachloro-1,1′,3,3′-tetraethylbenzimidazolylcarbocyanine iodide (JC-1). Briefly, cells were plated in 6-well plates at a density of 450,000 cells/well and after treatment the cells were incubated with 2.5 *μ*g/mL JC-1 in culture medium at 37°C for 20 min. After washing in PBS, cells were analyzed by flow cytometry with band-pass filters of 533/30 nm (FL1 channel) and 585/40 nm (FL2 channel), respectively. 30,000 events were collected for each sample. For detection of JC-1 dye, logarithmic signal amplification with typical green-orange electronic signal compensation near 4% and orange-green electronic signal compensation around 10% was used. ΔΨm (red/green JC-1 fluorescence) was expressed as percentage of the control.

### 2.11. Determination of Intracellular GSH Levels

Intracellular reduced glutathione (GSH) levels were determined as described by Hissin and Hilf [32]. The cells were seeded in 25 cm^2^ flask at a density of 1.2 × 10^6^ cells/flask. After treatment, the cells were harvested by centrifugation at 700 g for 10 min at 4°C, washed twice with ice-cold PBS, then suspended in 300 *μ*L ice-cold lysis buffer (1/2 (v/v) mixture of 1 mM hydrochloric acid/10% (v/v) phosphoric acid), and incubated on ice for 15 min. After centrifugation at 15,000 g for 15 min at 4°C, an aliquot of 12.5 *μ*L supernatant was diluted with 250 *μ*L of 0.1 M Na_2_HPO_4_ buffer (pH 8.0) containing 0.1% (w/v) EDTA and finally 20 *μ*L of this solution was added to 320 *μ*L derivatizing solution (1/15 (v/v) mixture of 0.1% (w/v) ortho-phthalaldehyde in methanol/0.1 M Na_2_HPO_4_ buffer (pH 8.0) containing 0.1% (w/v) EDTA). After incubation at RT for 15 min in the dark, the reaction mixture was analyzed by HPLC with Waters 60F pumps and 600 pumps control unit system equipped with a X-Bridge C18, 5 *μ*m, 4.6 × 150 mm column associated with a Symmetry C18, 3.9 × 20 mm guard column (Waters Corporation, Milford, Massachusetts, USA) and a Waters 71P autosampler. The mobile phase consisted of 15% (v/v) methanol in 25 mM Na_2_HPO_4_, pH 6.0. Isocratic elution was performed at 37°C at a flow rate of 0.5 mL/min. The excitation/emission wavelengths were set to 350/420 nm in a Shimadzu RF-551 spectrofluorometric detector. The instrument control and data acquisition were carried out using the Waters Millennium^32^ software. GSH intracellular levels, normalized for cell number, were expressed as a percentage compared to the control cells.

### 2.12. Real Time PCR Analyses

The cells were plated in 6-well plates at a density of 450,000 cells/well and after treatment total RNA for reverse transcription-polymerase chain reaction (RT-PCR) was extracted using Trizol reagent according to manufacturer's instructions. Reverse transcription was performed from 1 *μ*g of total RNA using the QuantiTect Reverse Transcription kit as recommended by the manufacturer. Real-time quantitative RT-PCR (qRT-PCR) measurements were performed using a Bio-Rad iCycler iQ (Bio-Rad, Milan, Italy) using ribosomal protein S27a (*RPS27A*) housekeeping gene as normalizing control. The genes of which we evaluated expression levels were heme oxygenase-1 (*HO-1*), heat shock protein 70 (*HSP70*) thioredoxin reductase (*TRXR*), and thioredoxin (*TRX*). For this purpose, we chose the following primers designed with GenScript Real-time PCR Primer Design Software:* HO-1*_for GTCAGAGGCCCTGAAGGAG and* HO-1*_rev GCCCTTCTGAAAGTTCCTCA,* HSP70*_for CAAGAAGAAGGTGCTGGACA and* HSP70*_rev TCCTCTTGTGCTCAAACTCG,* TRXR*_for GGTCCAAATGCTGGAGAAGT and* TRXR*_rev TGGATTCCAATTGTGCTGTC,* TRX*_for GCAGATCGAGAGCAAGACTG and* TRX*_rev CACGTGGCTGAGAAGTCAAC,* RPS27A*_for AAGCACCAAATTGATGGTCA and* RPS27A*_rev AACCACTGGGTCACATAATCC. The amplifications of qRT-PCR were monitored using the SYBR Green fluorescent stain and the presence of a single PCR product was verified by dissociation analysis in all amplifications. The comparative threshold cycle (ΔΔCT) method was used to calculate the relative amount of gene expression. Intracellular mRNAs levels were reported as fold induction compared to the 0 h control cells.

### 2.13. Western Blotting for Hsp70

After treatment, SH-SY5Y cells (4 × 10^6^) were lysed in 100 *μ*L RIPA buffer (50 mM Tris-HCl (pH 7.4), 150 mM NaCl, 1% NP-40, 0.1% SDS, 2 mM EDTA, 50 mM NaF, 0,5% sodium deoxycholate) containing 1 mM PMSF, 0.2 mM Na_3_VO_4_, and SIGMAFAST Protease Inhibitor Cocktail. The lysates were incubated on ice for 30 min and centrifuged at 12,000 g for 20 min at 4°C. The supernatants were collected and proteins quantification was performed using a Bradford Assay (BIO-RAD). Equal amounts of proteins (20 *μ*g) were separated on 12% SDS-PAGE and transferred to nitrocellulose membrane probed with the primary anti-Hsp70 mAb (1 : 1000) (Stressgen) or antiactin mAb (1 : 5000) (Alexis). The protein bands were visualized by ECL system (Millipore) according to the manufacturer's instructions. Densitometric analyses were performed with Image J software and normalized to the reference actin protein. The intracellular Hsp70 protein levels were expressed as a percentage compared to the control cells.

### 2.14. NAD(P)H:Quinone Oxidoreductase 1 (NQO1) Activity

Cells were seeded in 75 cm^2^ flask at a density of 3.5 × 10^6^ cells/flask. After treatment, the cells were washed with PBS and lysed in 250 *μ*L of ice-cold 20 mM Tris-HCl buffer (pH 7.4) containing 250 mM sucrose, 1 mM phenylmethanesulfonyl fluoride, and* cOmplete* Protease Inhibitor Cocktail by using the Potter-Elvejham homogenizing system. The homogenate was centrifuged at 15,000 g for 20 min at 4°C. NQO1 activity was carried out as previously described [33]. Briefly, the reaction mixture (1 mL) contained 50 mM sodium phosphate buffer (pH 7.4), 0.7 mg/mL bovine serum albumin (BSA), 40 *μ*M 2,6-dichlorophenolindophenol (DCPIP), and 40 *μ*g sample proteins. The reaction was started by adding nicotinamide adenine dinucleotide phosphate reduced form (*β*NADPH) at a final concentration of 0.3 mM. The decrease in absorbance due to reduction of DCPIP was monitored at 600 nm during the first 10 sec of the kinetics. Unspecific activity was determined by adding 10 *μ*M dicumarol to the reaction mixture before addition of *β*NADPH and subtracted to total activity. NQO1 activity was calculated as nmol of reduced DCPIP per min per mg of total protein by using an extinction coefficient of 21 × 10^−3 ^M × cm^−1^. The intracellular NQO1 activity levels were expressed as a percentage compared to the control cells.

### 2.15. Nrf2 Immunostaining

Cells were seeded on 18 × 18 mm glass coverslips in 6-well plates at a density of 5 × 10^5^ cells/well. After treatment the cells were fixed by incubating with 2% (w/v) formaldehyde in PBS and washed with PBS and permeabilized with 0.1% (v/v) Triton X-100 in PBS. After washing with PBS and finally with 0.05% (v/v) Tween 20 in PBS (PBST), the coverslips were exposed for 30 min to blocking buffer (5% (w/v) BSA in PBST) and then incubated overnight at 4°C with the rabbit anti-Nrf2 mAb (1 : 100 dilution) in 1% (w/v) BSA in PBST. The coverslips were washed with PBST and with PBS before incubation for 1 h with the secondary antibody Alexa Fluor 488 goat anti-rabbit IgG (1 : 500 dilution) in 1% (w/v) BSA in PBST. After washing with PBST and with PBS and incubating for 30 min with 5 *μ*g/mL of 4′,6-diamidino-2-phenylindole dihydrochloride (DAPI) in saline solution, the coverslips were washed again with PBS and mounted with 50% (v/v) glycerol in PBS. The samples were examined by fluorescence microscope (DM IL LED model, Leica Microsystems CMS Gmb H, Wetzlar, Germany) equipped with an 355 nm excitation filter, an 465 nm emission filter, and a digital camera (Leica DCF450 C). Microscopy imaging was performed using the LAS AF software. Control samples were treated following identical protocol but omitting the primary antibody.

To analyze the Nrf2 nuclear translocation by immunostaining assay, Image J software was used and 3 fields for each sample were analyzed. The average nuclear signal quantification was obtained by integrated density measurement for each nucleus subtracted of background signal, according to

(1) $$\begin{array}{c} \overset{N}{\underset{i=1}{\sum }}\frac{\text{IntDen}\_i-\left(A\_i*\langle \text{bk}\rangle \right)}{N}, \end{array}$$

where IntDen_*i* is the integrated density signal measured for each nucleus, *A*_*i* corresponds to single nucleus area, and 〈bk〉 features the mean of background signals. Densitometric analysis data were expressed as percentage of control.

### 2.16. Statistical Analysis

Experiments were repeated at least in triplicate and all the results are expressed as the mean value ± standard error of the mean (SEM). Statistical comparison between groups was made using unpaired Student's *t*-test. *P* values <0.05 were regarded as significant.

## 3. Results

### 3.1. Polyphenols and Sulforaphane Determination and Antioxidant Potential of Broccoli Sprouts Juices

The quantification of phenolics in the two broccoli sprouts juices shows a significant difference in bioactive phenols content between juice A (obtained from sprouts grown in the dark) and juice B (obtained from sprouts grown under light and sucrose stress). As shown in Table 1, juice B shows a 2-fold enrichment of total polyphenols compared to juice A (2.86 in B* versus* 1.40 in A mg GAE/mL, *P* < 0.05). Among polyphenols, flavonoid increase was found to be 2.7-fold (1.18 in B* versus* 0.44 in A mg RE/mL, *P* < 0.001), whereas anthocyanins, undetectable in juice A, were found to be 29.41 *μ*g CGE/mL in juice B. Conversely, a comparable content of SFN was observed in both juices (20.40 in B* versus* 18.60 in A *μ*g/mL, *P* > 0.05). To strengthen the evidence of the increase in polyphenols content, an evaluation of the intrinsic antioxidant activity of the juices was performed with several assays targeted versus different free radicals,* that is*, superoxide anion, ABTS radical cation, DPPH nitrogen radical, hydroxyl radical, and peroxyl radical (Table 2). Table 2 shows IC_50_ and ORAC values of the two juices, indicating that juice B shows a significantly higher antioxidant capability compared to juice A. The ~2-fold increase of antioxidant activity was in accordance with the enrichment in total phenolics amount.

### 3.2. Protective Effect of Broccoli Sprouts Juices on A*β*_25–35_-Induced Cytotoxicity

Preliminary investigations were carried out to determine the effect of juice A and juice B on the viability of SH-SY5Y cells, in order to verify that these mixtures did not exert a direct cytotoxic effect and to optimize the amount of juice to be used in further experiments. Both juices showed no negative effects on cell viability up to a final concentration of 10 *μ*L/mL and for 72 h of treatment. Juice B at higher concentrations (≥20 *μ*L/mL) was found to reduce cell viability in the long term (data not shown). Based on these results, the possible protective effect of both broccoli juices on the viability of the A*β*_25–35_-treated SH-SY5Y cells was studied at a final concentration of 10 *μ*L/mL. The final concentration of the various compounds in culture medium was around 1 *μ*M for sulforaphane for both juices and in the micromolar range for polyphenols, as inferred from Table 1.

**Figure 1:**
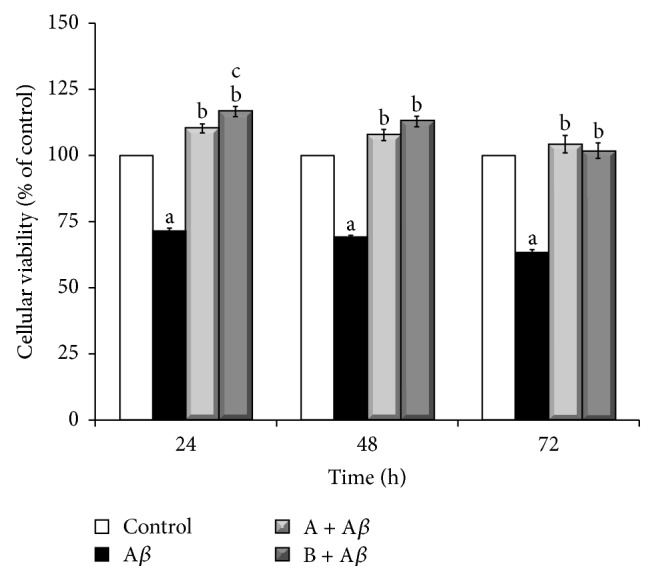
Protective effect of broccoli sprouts juices on A*β*_25–35_-induced cytotoxicity. SH-SY5Y cells were incubated with 25 *μ*M A*β*_25–35_ in the presence or in the absence of 10 *μ*L/mL of juice A or juice B for 24, 48, and 72 h. Cell viability was determined by MTT reduction assay and expressed as percentage compared to control. Data are represented as mean ± SEM (*n* = 5), ^a^ *P* < 0.001* versus* control; ^b^ *P* < 0.001* versus* A*β*_25–35_; ^c^ *P* < 0.05* versus* (A + A*β*).

As shown in Figure 1, 25 *μ*M A*β*_25–35_ treatment significantly decreased SH-SY5Y cells viability as compared to untreated cells (~70% at 24–48 h and 63% at 72 h, *P* < 0.001* versus* control). The cotreatment with 10 *μ*L/mL juice A or juice B significantly reduced A*β*_25–35_-induced cytotoxicity, restoring a cell viability comparable to that of the control (110% in (A + A*β*) and 117% in (B + A*β*)* versus* 71% in A*β* at 24 h, *P* < 0.001; 108% in (A + A*β*) and 113% in (B + A*β*)* versus* 69% in A*β* at 48 h, *P* < 0.001; 104% in (A + A*β*) and 102% in (B + A*β*)* versus* 63% in A*β* at 72 h, *P* < 0.001).

### 3.3. Protective Effect of Broccoli Sprouts Juices on A*β*_25–35_-Induced Apoptosis

**Figure 2:**
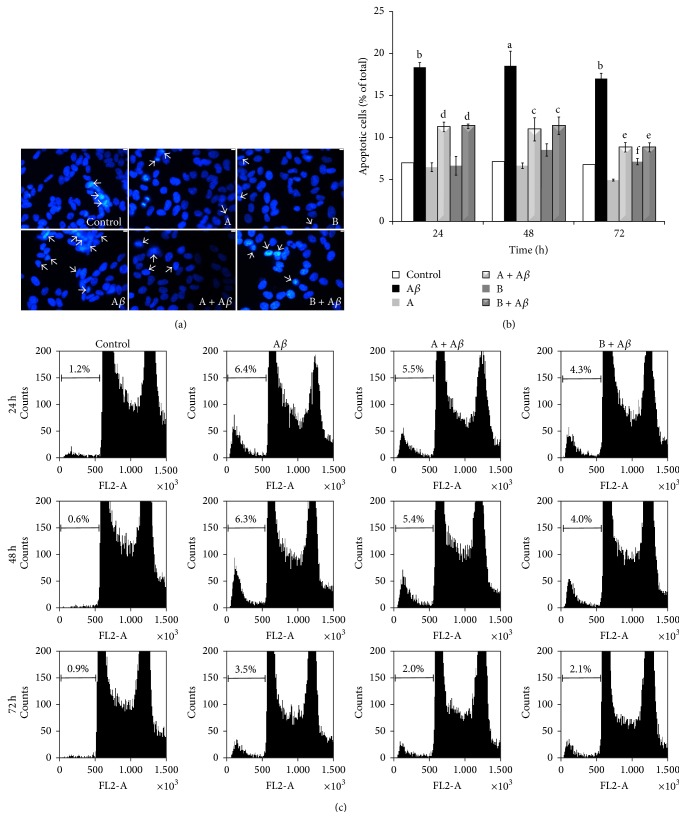
Protective effect of broccoli sprouts juices on A*β*_25–35_-induced apoptosis. SH-SY5Y cells were incubated with 25 *μ*M A*β*_25–35_ in the presence or in the absence of 10 *μ*L/mL of juice A or juice B for 24, 48, and 72 h. (a) Morphological analysis of apoptosis was achieved by staining of cell nuclei with Hoechst 33258. Arrows indicate the apoptotic cells. Images are representative of three different experiments. (b) Quantification of the percentage of apoptotic cells by fluorescence microscopy with the DNA intercalating Hoechst 33258. Data are expressed as mean ± SEM (*n* = 3), ^a^ *P* < 0.05 and ^b^ *P* < 0.001* versus* control; ^c^ *P* < 0.05, ^d^ *P* < 0.01, and ^e^ *P* < 0.001* versus* A*β*_25–35_; ^f^ *P* < 0.05* versus* juice A. (c) Analysis by flow cytometry of the sub-G1 peak in cells stained with propidium iodide (PI). The percentage of cells with hypodiploid DNA content is shown as function of the PI fluorescence signal (FL2-A). Histograms are representative of three independent experiments.

SH-SY5Y cells apoptosis was evaluated by fluorescence microscopy by using the nuclear dye Hoechst 33258 (Figure 2(a)). Treatment of cells with 10 *μ*L/mL of juice A or juice B showed no proapoptotic effect compared to controls, whereas apoptosis increased from 7% in control cultures to about 18% in cells treated with 25 *μ*M A*β*_25–35_ for 24 (*P* < 0.001), 48 (*P* < 0.05), and 72 h (*P* < 0.001). Following treatment of the cells with A*β*_25–35_ in the presence of 10 *μ*L/mL of juice A or juice B, a significant reduction in apoptotic cells was observed compared to the treatment with A*β*_25–35_ alone (11% in the cotreatment with juice A or juice B and A*β*_25–35_ *versus* 18% in the exposure to A*β*_25–35_ alone at 24 (*P* < 0.01) and 48 h (*P* < 0.05); 9% in the cotreatment with juice A or juice B and A*β*_25–35_ *versus* 18% in exposure to A*β*_25–35_ alone at 72 h (*P* < 0.001) (Figure 2(b)). To confirm these data flow cytometric analyses were also performed by staining treated cells with PI (Figure 2(c)). Data obtained revealed that 25 *μ*M A*β*_25–35_ treatment induced a marked increase in the sub-G1 (hypodiploid) fraction of the total cell population, compared to the control group with percentages raising from 1.2% to 6.4% at 24 h, from 0.6% to 6.3% at 48 h, and from 0.9% to 3.5% at 72 h. Flow cytometry analysis confirmed the absence of any proapoptotic effect of both juice A and juice B (data not shown). Treatment of cells with 25 *μ*M A*β*_25–35_ in the presence of 10 *μ*L/mL of juice A or juice B reduced apoptotic cells fraction from 6.4% in A*β* to 5.5% in (A + A*β*) and to 4.3% in (B + A*β*) at 24 h; from 6.3% in A*β* to 5.4% in (A + A*β*) and to 4.0% in (B + A*β*) at 48 h; finally, from 3.5% in A*β* to 2.0% in (A + A*β*) and to 2.1% in (B + A*β*) at 72 h.

### 3.4. Broccoli Sprouts Juices Counteract A*β*_25–35_-Induced Reduction of the ΔΨm

**Figure 3:**
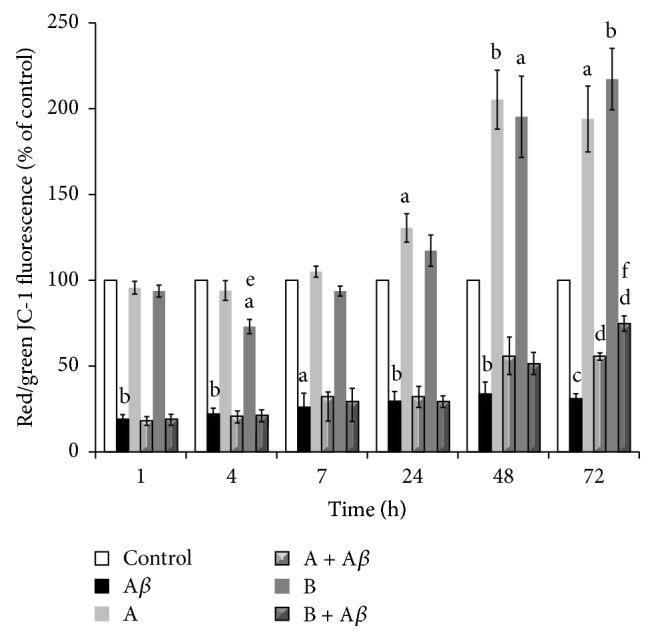
Effect of broccoli sprouts juices on A*β*_25–35_-induced mitochondrial membrane potential (ΔΨm) depletion. SH-SY5Y cells were incubated with 25 *μ*M A*β*_25–35_ in the presence or in the absence of 10 *μ*L/mL of juice A or juice B for 1, 4, 7, 24, 48, and 72 h. After treatment the cells were stained with JC-1 and analyzed by flow cytometry. The ΔΨm is proportional to the ratio between red and green fluorescence of the mitochondrial probe JC-1 and is expressed as a percentage of the control. Data are represented as mean ± SEM (*n* = 3), ^a^ *P* < 0.05, ^b^ *P* < 0.01, and ^c^ *P* < 0.001* versus* control; ^d^ *P* < 0.001* versus* A*β*_25–35_; ^e^ *P* < 0.05* versus* juice A; ^f^ *P* < 0.05* versus* (A + A*β*).

We also confirmed the protective effect of broccoli sprouts juices against A*β*_25–35_-induced apoptotic cell death by examining a proapoptotic signal such as the reduction of the ΔΨm. Cells exposed to 25 *μ*M A*β*_25–35_ showed a rapid reduction in ΔΨm (Figure 3), which at 1 and 4 h of incubation was found to be ~20% compared to control cells (*P* < 0.01), whereas starting from 7 h up to 72 h it was ~30% (statistical significance ranging from *P* < 0.05 to *P* < 0.001). During the first 7 h of treatment with 10 *μ*L/mL of juice A, no significant changes in ΔΨm were observed whereas a marked and progressive increase for longer exposure times was evidenced, with ΔΨm values reaching 205% of control at 48 h (*P* < 0.01) and 194% of control at 72 h (*P* < 0.05). When the cells were incubated with 10 *μ*L/mL of juice B, a decrease of ΔΨm (73%* versus* control, *P* < 0.05) was observed after 4 h of exposure, but on the other hand the ΔΨm readily returned to control values within 7 h of treatment. Similarly to what was observed for juice A, also juice B induced a significant increase of ΔΨm, from 48 h of exposure (195%, *P* < 0.05) and up to 72 h (217%, *P* < 0.01), compared to control cells. When the cells were treated with A*β*_25–35_ in the presence of juice A or juice B, no protective effect on the ΔΨm was observed within 48 h of treatment, whereas after 72 h of incubation, broccoli sprouts juices significantly improved A*β*_25–35_-induced impairment in ΔΨm, with juice B being more effective than juice A (75%* versus* 56%, resp., *P* < 0.05).

### 3.5. Broccoli Sprouts Juices Downregulate A*β*_25–35_-Mediated Apoptotic Cell Death Signalling Pathways by Inducing the HSP70 Gene Overexpression

**Figure 4:**
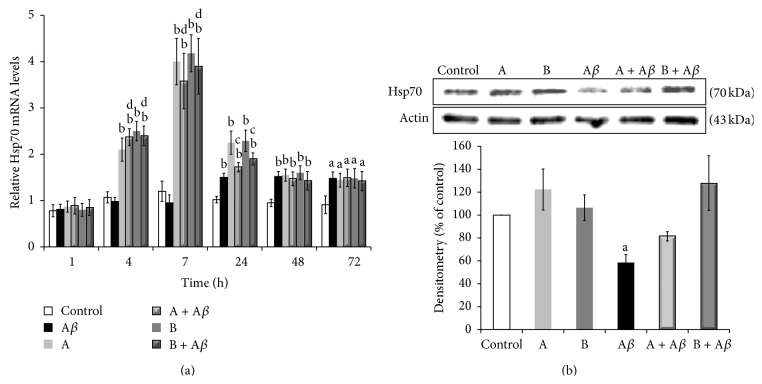
Broccoli sprouts juices protect SH-SY5Y cells from A*β*_25–35_-mediated apoptosis through upregulation of* HSP70* gene expression. (a) SH-SY5Y cells were incubated with 25 *μ*M A*β*_25–35_ in the presence or in the absence of 10 *μ*L/mL of juice A or juice B for 1, 4, 7, 24, 48, and 72 h. After treatment total RNA was extracted and used for qRT-PCR determination of* HSP70* gene induction. The* RSP27A* housekeeping gene was used as a control for normalization of the data. The relative amounts of gene expression were calculated by the comparative threshold cycle (ΔΔCT) method. The intracellular levels of mRNA are expressed as fold induction compared to control cells at 0 h of incubation. (b) Cell lysates obtained after 7 h of treatment were subjected to western blot analysis with anti-Hsp70 antidoby. Actin levels were compared to ensure equal amount of protein loading. The intracellular Hsp70 protein levels in the samples were expressed as a percentage compared to the control. Data are represented as mean ± SEM (*n* = 3), ^a^ *P* < 0.05 and ^b^ *P* < 0.01* versus* control; ^c^ *P* < 0.05 and ^d^ *P* < 0.01* versus* A*β*_25–35_.

To elucidate the molecular mechanisms underlying the antiapoptotic effects of broccoli sprouts juices in SH-SY5Y cells, expression of the* HSP70* gene was examined. The heat shock protein Hsp70 plays a key role in the cellular response for the protection from oxidative stress [34–36]. The determination by qRT-PCR of Hsp70 mRNA levels in SH-SY5Y cells (Figure 4(a)) showed no significant changes upon exposure to 25 *μ*M A*β*_25–35_. Conversely, both broccoli sprouts juices, either in the absence or in the presence of A*β*_25–35_, induced an increase of* HSP70* gene expression starting from 4 h of incubation and with a peak at 7 h (3.5–4 times* versus* control and A*β*_25–35_, *P* < 0.01), still persistent up to 24 h.

The protein levels of Hsp70, as measured by western blot analysis (Figure 4(b)), were found to be significantly decreased after 7 h of treatment with A*β*_25–35_, in line with literature data [37]. The cotreatment with both juice A and juice B restored Hsp70 protein levels, highlighting the importance of Hsp70 as an antiapoptotic factor.

### 3.6. Protective Effect of Broccoli Sprouts Juices on A*β*_25–35_-Induced GSH Depletion

**Figure 5:**
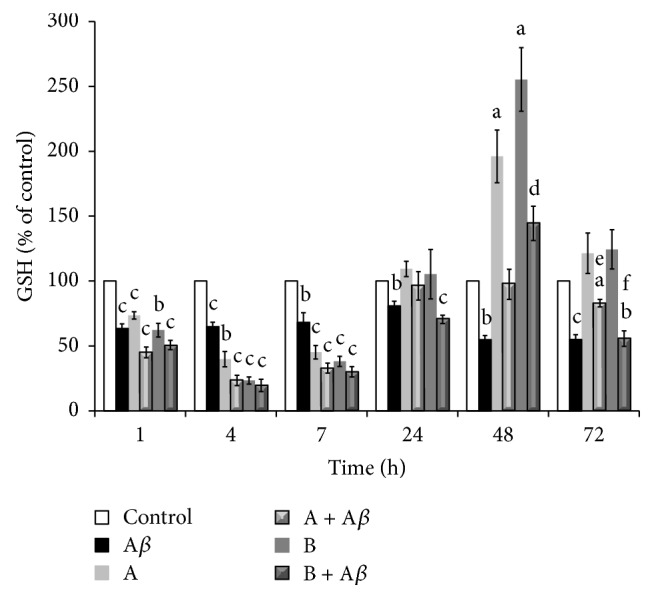
Protective effect of broccoli sprouts juices from A*β*_25–35_-induced GSH depletion. SH-SY5Y cells were incubated with 25 *μ*M A*β*_25–35_ in the presence or in the absence of 10 *μ*L/mL of juice A or juice B for 1, 4, 7, 24, 48, and 72 h. After treatment, total cell lysate was prepared for the determination of intracellular GSH levels by RP-HPLC analysis, using the fluorescence signal of the derivatized product with OPA. The concentration of GSH in the samples, normalized by the number of cells, was expressed as a percentage compared to the control. Data are represented as mean ± SEM (*n* = 3–5) ^a^ *P* < 0.05, ^b^ *P* < 0.01, and ^c^ *P* < 0.001* versus* control; ^d^ *P* < 0.05 and ^e^ *P* < 0.01* versus* A*β*_25–35_; ^f^ *P* < 0.05* versus* (A + A*β*).

It is well known that A*β* can induce oxidative stress* via* several different mechanisms thus triggering cell death [38]. To further verify whether A*β*_25–35_ treatment induces oxidative stress in our model, we determined the intracellular content of GSH, one of the major antioxidant defence systems in the cell. As shown in Figure 5, the treatment of SH-SY5Y cells with 25 *μ*M A*β*_25–35_ up to 72 h was responsible for a rapid and significant reduction of intracellular GSH levels compared to the control group, respectively, of ~65% in the first 7 h of incubation, 81% after 24 h, and finally 55% after 48 h, up to 72 h of exposure. Both juice A and juice B, also determined a fast and significant decrease in the intracellular GSH levels compared to control cells, with a* minimum* achieved within 4 h of treatment (40% and 23% for juice A and juice B, resp.). On the other hand, after 7 h of incubation, and even more clearly within 24 h of exposure to the juices, a cellular response was observed that led to the progressive recovery of GSH levels comparable to those of the control. Notably, treatment for 48 h with the juices induced a substantial increase of the cellular content of GSH, 196% for juice A (*P* < 0.05) and 255% for juice B (*P* < 0.05) compared to control, which returned to basal levels within 72 h. The treatment of SH-SY5Y cells with 25 *μ*M A*β*_25–35_ in the presence of 10 *μ*L/mL of juice A or juice B showed a synergic effect on intracellular GSH depletion, reaching a* minimum* at 4 h of incubation (~20%, compared to the controls) and a recovery after 7 h of cotreatment. Within 48 h of exposure to A*β*_25–35_ and the juices, the intracellular GSH content recovered to the level of the control group in the presence of juice A (97%) and even increased up to 144% with the juice B (*P* < 0.05* versus* A*β*_25–35_). In prolonged incubations up to 72 h, a protective effect on A*β*_25–35_-mediated depletion of intracellular GSH content was still observed in the presence of juice A (83%* versus* 55% for A*β*_25–35_, *P* < 0.05), while for the juice B such capacity was not appreciable (56%* versus* 55% for A*β*_25–35_, *P* > 0.05).

### 3.7. Broccoli Sprouts Juices Upregulated Cellular Antioxidant Defence Capacity via Activation of Nrf2

**Figure 6:**
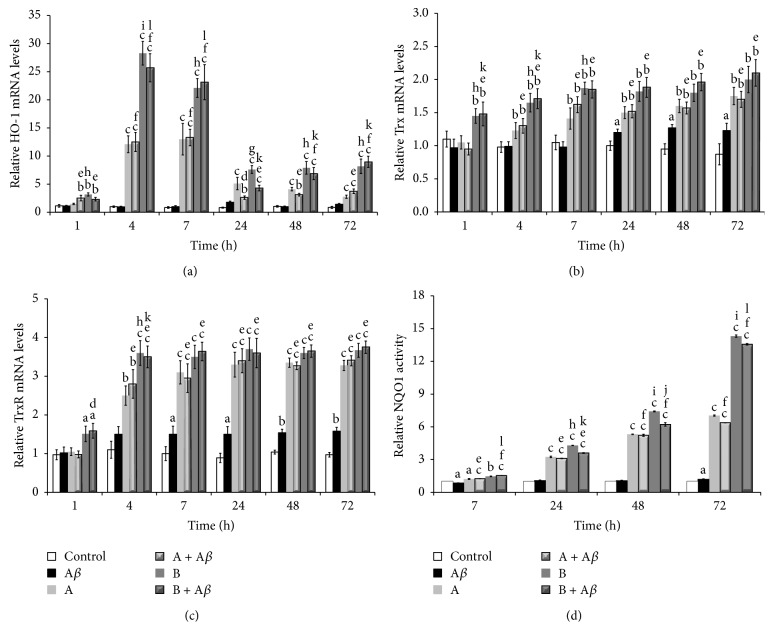
Broccoli sprouts juices upregulate the cellular antioxidant defence capacity. SH-SY5Y cells were incubated with 25 *μ*M A*β*_25–35_ in the presence or in the absence of 10 *μ*L/mL of juice A or juice B for 1, 4, 7, 24, 48, and 72 h. After treatment, total RNA was extracted and used for qRT-PCR determination of gene expression of (a)* HO-1*; (b)* TRX*; (c)* TrxR*. The* RSP27A* housekeeping gene was used as a control for normalization of the data. The relative amounts of gene expression were calculated by the comparative threshold cycle (ΔΔCT) method. The intracellular levels of mRNA are expressed as fold induction compared to control cells at 0 h of incubation. (d) Total cell lysates were subjected to spectrophotometric assay of NQO1 activity which was expressed as a percentage compared to the control. Data are represented as mean ± SEM (*n* = 3), ^a^ *P* < 0.05, ^b^ *P* < 0.01, and ^c^ *P* < 0.001* versus* control; ^d^ *P* < 0.05, ^e^ *P* < 0.01, and ^f^ *P* < 0.001* versus* A*β*_25–35_; ^g^ *P* < 0.05, ^h^ *P* < 0.01, and ^i^ *P* < 0.001* versus* juice A; ^j^ *P* < 0.05, ^k^ *P* < 0.01, and ^l^ *P* < 0.001* versus* (A + A*β*).

To investigate the molecular mechanisms of neuroprotection exerted by broccoli sprouts juices against A*β*_25–35_-induced cell death, the expression or activity of various cellular antioxidant enzymes was examined. For this purpose, we determined the mRNA levels of enzymes such as HO-1, Trx, and TrxR by qRT-PCR. Moreover, we tested the activity of the enzyme NQO1 by a specific spectrophotometric assay. As shown in Figure 6(a),* HO-1* gene expression in SH-SY5Y cells treated with 25 *μ*M A*β*_25–35_ for 1, 4, 7, 24, 48, and 72 h showed little fluctuation. In contrast, following exposure to 10 *μ*L/mL of juices A or B, either alone or in the presence of 25 *μ*M A*β*_25–35_, HO-1 mRNA levels in the cells were strongly increased. Relative to control group, HO-1 mRNA started to increase after 4 h and up to 7 h treatment with broccoli sprouts juices. In this time frame,* HO-1* gene expression increased ~12 times (*P* < 0.001) with juice A and 25 times (*P* < 0.001) with juice B compared to control, respectively. At 24, 48, and 72 h of exposure, HO-1 mRNA levels were substantially lower than at 4 h, although an appreciable* HO-1* gene induction was still present compared to control cells. All through the time points, juice B was significantly more effective in upregulating* HO-1* gene expression compared to juice A, both in the absence and in the presence of A*β*_25–35_. Trx mRNA levels were altered as a result of exposure to A*β*_25–35_ only from 24 h, remaining constant up to 72 h (Figure 6(b)). In any case, the magnitude of induction was rather weak (1.2 times* versus* control, *P* < 0.05). Treatment with broccoli sprouts juices, alone or in presence of A*β*_25–35_, significantly increased* TRX* gene expression starting from 1 h of incubation with juice B and at 4 h with juice A. Compared to control, during the first 7 h of exposure, the increase of Trx mRNA levels observed with juice B was higher than that caused by juice A, which only within 24 h, and up to 72 h, matched the inductive capacity of juice B. The* maximum* increase in* TRX* gene expression was observed at 72 h of treatment with broccoli sprouts juices, either in the absence or in the presence of A*β*_25–35_, with levels reaching 1.7 and 2.0 times (*P* < 0.01) that of the control for juice A and juice B, respectively. The pattern of the kinetics of* TRXR* gene expression was comparable to that of the above described* TRX* gene (Figure 6(c)). After 7 h of incubation, and up to 72 h, the treatment with 25 *μ*M A*β*_25–35_ determined a modest (1.5 times) and stable increase in the level of TrxR mRNA compared to the control group (*P* < 0.05 at 7 and 24 h; *P* < 0.01 at 48 and 72 h). Already after 1 h of exposure, 10 *μ*L/mL of juice B alone or in the presence of A*β*_25–35_ induced a significant increase (1.5 times* versus* control, *P* < 0.05) of* TRXR* gene expression levels which reached a* maximum* (3.6 times* versus* control, *P* < 0.05) at 4 h and kept constant up to 72 h. The induction of* TRXR* gene by juice A was evidenced only after 4 h of treatment, both in the absence and in the presence of A*β*_25–35_ (~2.6 times* versus* control, *P* < 0.01), reaching a plateau within 24 h (~3.3 times* versus* control, *P* < 0.001). At 7 h of exposure and for longer incubation periods, the effect of juice A was comparable to that of juice B. Measurement of NQO1 activity levels in SH-SY5Y cells did not show any effect of A*β*_25–35_ compared to control (Figure 6(d)). Otherwise, starting from 7 h of treatment with broccoli sprouts juices, either alone or in the presence of A*β*_25–35_, the NQO1 activity showed a time-dependent increase, with a significantly higher effect of juice B compared to juice A. The stimulating effect of juice B on NQO1 activity was higher than that of juice A from 24 h up to 72 h of exposure (24 h: 3.1 times A and (A + A*β*)* versus* control, *P* < 0.001 and 3.9 times B and (B + A*β*)* versus* control, *P* < 0.001; 48 h: 5.2 times A and (A + A*β*)* versus* control, *P* < 0.001 and 6.8 times B and (B + A*β*)* versus* control, *P* < 0.001; 72 h: 6.7 times A and (A + A*β*)* versus* control, *P* < 0.001 e 13.9 times B and (B + A*β*)* versus* control, *P* < 0.001).

**Figure 7:**
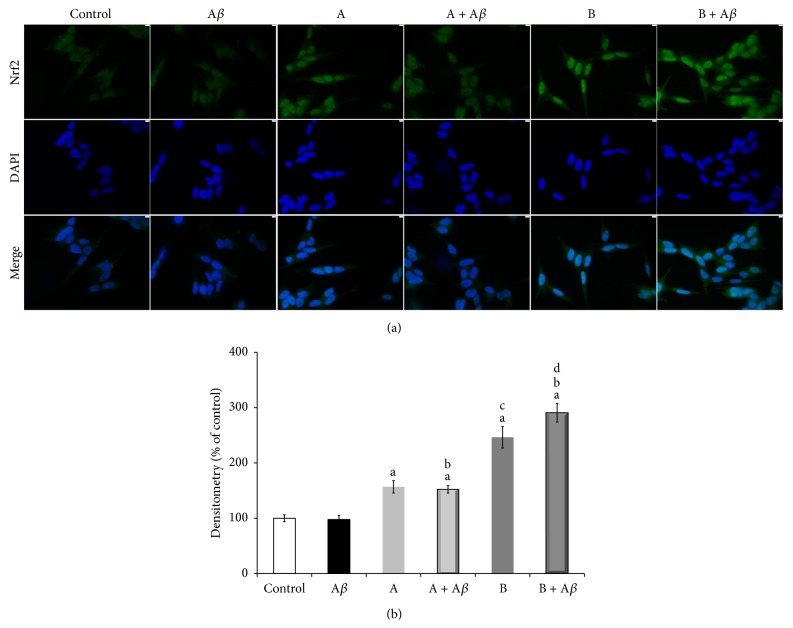
Broccoli sprouts juices activate nuclear translocation of Nrf2 transcription factor which controls the expression of cell survival genes involved in defence against oxidative stress-mediated damage. SH-SY5Y cells were incubated with 25 *μ*M A*β*_25–35_ in the presence or in the absence of 10 *μ*L/mL of juice A or juice B for 3 h. (a) Nrf2 nuclear translocation was verified by immunofluorescence microscopy by utilizing anti-Nrf2 antibody and DAPI staining. Representative images of three independent experiments show the stimulation of Nrf2 nuclear accumulation by broccoli sprouts juices A or B exposure, compared to both control cells and A*β*_25–35_ alone treated cells. (b) Densitometric analysis of the mean nuclear located-Nrf2 fluorescence signal as calculated analyzing immunofluorescence images by the Image J software. Data are expressed as percentage of control and represented as mean ± SEM (*n* = 3), ^a^ *P* < 0.001* versus* control; ^b^ *P* < 0.001* versus* A*β*_25–35_; ^c^ *P* < 0.001* versus* juice A; ^d^ *P* < 0.001* versus* (A + A*β*).

To elucidate the upstream signalling pathway involved in the broccoli sprouts juices-induced upregulation of the antioxidant enzymes, we focused on the activation of the redox-sensitive transcription factor Nrf2. When SH-SY5Y cells were treated with A*β*_25–35_, or with juices A or B, alone or in the presence of A*β*_25–35_, differences in Nrf2 nuclear translocation between samples after 3 h of incubation were observed (Figures 7(a) and 7(b)). In particular, densitometric analysis of the images collected by immunofluorescence microscopy showed that, compared to control cells, the nuclear content of Nrf2 was not altered by A*β*_25–35_, while both broccoli sprouts juices were able to induce nuclear translocation of Nrf2, both alone (157% A* versus* control, *P* < 0.001; 246% B* versus* control, *P* < 0.001) and in the presence of A*β*_25–35_ (152% (A + A*β*)* versus* control and A*β*_25–35_, *P* < 0.001; 291% (B + A*β*)* versus* control and A*β*_25–35_, *P* < 0.001), with juice B being significantly more effective than juice A.

## 4. Discussion

Epidemiological studies have highlighted the capacity of* Brassica* species to prevent cardiovascular diseases as well as to counteract the onset and progression of certain types of tumours [39, 40]. A number of clinical trials have shown favorable effects on oxidative stress and an improvement of insulin resistance in type 2 diabetes patients that consumed broccoli sprouts [41, 42]. Oxidative stress is recognized as a common factor in many neurodegenerative diseases [43], and the identification of novel antioxidants as potential therapeutics is a prolific area of neuroscience research [44].

Although several studies on biological systems, both* in vitro* and* in vivo*, have been devoted to the evaluation of the neuroprotective effects of single, purified bioactive components of plant foods, for example, polyphenolic compounds [16, 18] or organosulfur compounds such as SFN [14, 17], less investigated are food matrices as a whole, where many of these molecules interact in a complex mixture. The present study utilized broccoli sprouts crude juices with a different antioxidant phytochemicals profile to investigate their potential role as antioxidants and neuroprotective agents. Differences in phytochemical content in broccoli sprouts juices allowed us to investigate the relationship between phenolic content, antioxidant capacity, and protection of neuronal cells in culture from oxidative damage, in particular in the AD cell model represented by SH-SY5Y cell line exposed to A*β*_25–35_, one of the most toxic fragments of the full length peptide A*β*_1–42_. In addition, our findings contribute to the elucidation of the molecular mechanisms involved in the neuroprotective action of polyphenols, evidencing the relevance of the cell survival signalling pathways regulated by the transcription factor Nrf2.

The germination of broccoli seeds in different growth conditions (in the dark or under white light in the presence of sucrose) enabled us to obtain two groups of seedlings that provided, by cold pressing, raw juices with significantly different phenolics content. The juice B obtained from sprouts grown under stress conditions was enriched in total polyphenols, flavonoids, and in particular anthocyanins with respect to juice A. Our data are in line with previous investigations by Pérez-Balibrea et al. [45] and by Guo et al. [46] regarding the effect of light and sucrose stress on the modulation of phenolic content in broccoli sprouts.

As polyphenols are known to possess antioxidant activity, the intrinsic antioxidant potential of the two juices was tested by means of various assays specific for different species of free radicals. Results pointed to ~2-fold higher intrinsic antioxidant properties of juice B compared to juice A, in line with the different total polyphenol content of the two juices that were found to be ~2-fold higher in juice B than in juice A. These data indicate that the procedures adopted to alter the content of phenolic compounds in broccoli sprouts were successful in significantly increasing the levels of polyphenols, and in particular anthocyanins, leading to a proportional difference in the overall antioxidant capacity.

The extra- and intraneuronal aggregation and deposition of A*β* play a causal role in the pathogenetic cascade leading to AD [47, 48]. A*β* oligomers adhere to the plasma membrane of neurons and cause neuronal death* via* a combination of free radical damage and formation of ion-permeable pores [49]. Excessive production of reactive oxygen species (ROS) by A*β* peptides and exhaustion of the endogenous antioxidant defence systems including GSH, catalase, superoxide dismutase, and glutathione metabolizing enzymes can cause oxidative damages to critical cellular macromolecules, mitochondrial dysfunction, and altered cellular signal transduction cascades [50]. Given the antioxidant capability of juices A and B, we examined their protective effects against A*β*_25–35_-induced neurotoxicity in SH-SY5Y cells. The cotreatment of SH-SY5Y cells with 25 *μ*M A*β*_25–35_ and 10 *μ*L/mL juice A or juice B led to a significant decrease of A*β* toxicity. At the juice concentration utilized, the amounts of polyphenols and of sulforaphane were in the low micromolar range, in line with literature data indicating that these compounds reach a similar concentration in plasma after oral ingestion [51].

SH-SY5Y cells treated with A*β*_25–35_ underwent apoptosis, as shown by alterations in nuclear morphology and hypodiploid cellular subpopulation levels and by perturbation of the mitochondrial transmembrane potential. However, cotreatment with broccoli sprouts juice effectively ameliorated the A*β*_25–35_-induced proapoptotic signs. Apparently, the difference between juices A and B in terms of intrinsic antioxidant activity observed in cell-free chemical systems is not reflected in significant differences in the overall neuroprotective effect against A*β*_25–35_-induced cytotoxicity. Hence the observed protective effect cannot be attributed to the direct antioxidant action of the extract, but most probably to other mechanisms still to be defined. For instance, Tarozzi et al. observed that the neuroprotective effect of the anthocyanin compound cyanidin-3-*O*-glucoside (Cy-3G) on A*β*_25–35_-induced toxicity in SH-SY5Y cells was mainly due to the ability of Cy-3G to hinder the adsorption of A*β*_25–35_ oligomers to the plasma membrane as well as to prevent cellular membrane and redox status impairment [52]. These authors also emphasize that the intrinsic antioxidant properties of Cy-3G may play a marginal role in its protective effects and showed the absence of Cy-3G antioxidant activity in the cytosol of SH-SY5Y cells, suggesting a low uptake of Cy-3G [53]. Actually, the anthocyanins content of juice A is undetectable and in juice B is such that the final concentration of these active compounds in culture medium reached under our experimental conditions was at least 100 times below those adopted in other studies on the neuroprotective activity of purified anthocyanins; hence the contribution of these class of compounds to the overall observed effect is doubtful.

Flavonoids exert cooperative effects by inhibiting distinct targets responsible for the generation of ROS and A*β*-induced toxicity, particularly at the mitochondrial level. Notably, complex I activity is reduced in patients with AD and PD, and excessive generation of ROS produced by neurotoxins such as rotenone and 1-methyl-4-phenylpyridinium (MPP+) is mediated by complex I inhibition [54]. It has been observed that quercetin binds to complex I preventing rotenone-induced production of superoxide anion with an IC_50_ = 1.8 *μ*M, without interfering with mitochondrial respiration up to a* maximum* of 10 *μ*M [55]. This indicates that flavonoids can behave as “coenzyme Q-mimetic molecules” allowing normal electron flow along the entire electron transport chain (ETC) [56]. Since in our experimental settings the final concentrations of flavonoids in culture medium are in the micromolar range, we hypothesize that the observed functional recovery of the ETC could be ascribed to the flavonoid fraction of the juices through the activation of the pathways mentioned above. This could also explain the observed mitochondrial hyperpolarization in cells treated with both juices. However, in the presence of A*β*_25–35_, broccoli sprouts juices slowly and only partially restore the ΔΨm to control values. A possible explanation could be that, despite the positive effect of flavonoids on the activity of the ETC, the mitochondrion is seriously damaged by A*β*_25–35_-induced mitochondrial permeability transition pore (PTP) opening responsible for a substantial dissipation of the protonic transmembrane gradient [50].

In addition to flavonoids, SFN is the component of the broccoli sprouts juices expected to exert a protective effect against A*β*_25–35_-mediated mitochondrial damage. Indeed, intraperitoneal injection of rats with a nontoxic level of SFN resulted in resistance of isolated nonsynaptic brain mitochondria to peroxide-induced PTP opening [57]. Our data indicate that broccoli sprouts juices confer protection of cellular bioenergetics and counteract the ΔΨm fall induced by A*β*_25–35_. These results are in agreement with a recent study by Lee et al. who evaluated the protective action of 5 *μ*M SFN against the ΔΨm fall induced by treatment with 15 *μ*M A*β*_25–35_ in SH-SY5Y cells [58].

We also investigated a possible molecular mechanism underlying the antiapoptotic effect of broccoli sprouts juices on SH-SY5Y cells treated with A*β*_25–35_. Literature data indicate that in response to various types of central nervous system injuries, including stroke, trauma, or neurodegenerative disorders, the heat-shock proteins Hsp70s are expressed and appear to have neuroprotective actions [36]. Patel et al. [34] reported that Hsp70 overexpression attenuated neuronal apoptosis in an* in vitro* model of amyotrophic lateral sclerosis. It has recently been suggested that in PD the neuroprotective effect of Hsp70 is due to not only its role as a chaperone that attenuates protein aggregation and toxicity but also a more direct antiapoptotic effect [36]. In particular, examining primary cortical neurons and the SH-SY5Y neuronal cell line transfected with* HSP70*-expression plasmids and subjected to four independent models of apoptosis, including A*β*_25–35_, Sabirzhanov et al. observed that Hsp70 overexpression corresponded to a significantly reduced induction of nuclear apoptotic markers and/or cell death [35]. Their results suggested that Hsp70 inhibits apoptosis both by attenuation of caspase-independent pathways by interacting with apoptosis-inducing factor (AIF) and preventing its translocation into the nucleus and by inhibiting caspase-dependent pathways by interacting with apoptotic protease-activating factor 1 (Apaf-1) thus blocking caspases activation. Our data demonstrate that broccoli sprouts juices induce an early overexpression of Hsp70 mRNA in the SH-SY5Y cell line, both in the absence and in the presence of A*β*_25–35_. Therefore, the broccoli sprouts juices may protect neurons from A*β*_25–35_-induced apoptosis through upregulation of the* HSP70* gene thus maintaining Hsp70 protein levels, allowing the inhibition of both caspase-dependent and caspase-independent programmed cell death pathways.

Cells possess a complex network of nonenzymatic and enzymatic components to counteract oxidative stress that are regulated by a common mechanism that involves two proteins: Nrf2 and the Kelch-like-ECH-associated protein 1 (Keap1). Nrf2 is normally sequestered in the cytoplasm by Keap1 which contains several reactive cysteine residues serving as sensors of the intracellular redox state. Upon oxidative or covalent modification of Keap1 cysteine residues, Nrf2 is released from Keap1 and translocates to the nucleus where it heterodimerizes with small musculoaponeurotic fibrosarcoma (Maf) proteins before binding to the Nrf2-antioxidant response element (ARE) within the promoter regions of the abovementioned cytoprotective genes [59, 60]. Polyphenols and organosulfur compounds such as SFN share the ability to activate Nrf2-ARE transcriptional pathway thus modulating the expression of antioxidant enzymes (HO-1, glutathione peroxidase (GPx), *γ*-glutamylcysteine ligase (GCL), glutathione reductase (GR), Trx, TrxR, etc.) and phase II xenobiotic detoxification enzymes (glutathione S-transferase (GST), NQO1, etc.). Polyphenols and SFN have an electrophilic centre that serves as an attack site for nucleophiles, such as specific protein sulfhydryl groups present on Keap1 and critical for Nrf2-Keap1 interaction [14, 16, 17]. Our immunofluorescence data revealed that the juices treatment determined the translocation of Nrf2 into the nucleus, demonstrating a consistent modulation of the Nrf2-ARE-dependent cell survival response. Notably, the greater effect was shown by polyphenols-enriched juice B, indicating that the polyphenol fraction of the complex food matrix has a leading role in the induction of the antioxidant response.

Since the Nrf2 activation is able to regulate GSH through the modulation of GSH-related enzymes we evaluated GSH level following sprout juice treatment. In our study the A*β*_25–35_-induced redox status of SH-SY5Y cells, in the presence or in the absence of broccoli sprouts juices, was evaluated by measuring cellular GSH levels. As expected, the oxidative stress associated with the A*β* peptide treatment, caused a significant reduction in the intracellular GSH content which persisted up to 72 h of incubation. During early stages of exposure, also broccoli sprouts juices, either alone or in combination with A*β*_25–35_, determined a conspicuous depletion of cellular GSH. This phenomenon is most probably due to conjugation of GSH with ITCs, such as SFN, present in the broccoli juices, and to the subsequent transformation of the glutathione dithiocarbamate conjugate to mercapturic acid, the major route of metabolism of ITCs in eukaryotic cells [61]. The activation of Nrf2 by polyphenols or by free ITCs escaped to glutathionylation may explain the increase of intracellular GSH levels via the induction of GCL observed after 24 h of treatment. In addition, the higher effect of juice B compared to juice A in counteracting A*β*_25–35_-mediated decrease of intracellular GSH in SH-SY5Y cells cotreated for 48 h can be attributed to the polyphenols enrichment of juice B compared to juice A. Actually, several studies indicate that polyphenols activate the transcription factor Nrf2 and increase the expression of the Nrf2 target gene* GCL* [62, 63]. However, after 72 h of treatment the increase in GSH intracellular content underwent a significant reduction, returning to basal levels in cells treated with the juices and lower than controls in cells treated with A*β*_25–35_ in the presence of the juices. It can be hypothesized that this phenomenon is the consequence of the overexpression of antioxidant enzymes such as GST, GPx, and NQO1,* via* the Nrf2-ARE transcriptional pathway activation mediated by SFN and polyphenols. Indeed, on the one hand GST and GPx determine a direct consumption of GSH for their enzymatic activity, and on the other hand, indirectly, NQO1, reducing quinones to semiquinones, subtracts NADPH necessary for the regeneration of GSH from GSSG by the enzyme GR. By investigating the mRNA expression or activity profiles of antioxidant enzymes known for their protective role against neurodegenerative disorders, we observed that broccoli sprouts juices, alone or in the presence of A*β*_25–35_, markedly increased the mRNA levels or activity of these enzymes in a time-dependent manner, compared to control and A*β*_25–35_-treated cells. In particular, between 4 and 7 h of incubation both juice A and juice B showed a* maximum* effect in the induction of the expression of* HO-1* gene, with juice B showing approximately twice the effect of juice A. Moreover, we observed a time-dependent increase of NQO1 activity caused by broccoli sprouts juices, compared to control and A*β*_25–35_-treated cells, the effect juice B being two times higher than that of juice A within 72 h of treatment, in line with the polyphenol content.

HO-1 catalyzes the stepwise degradation of heme to release free iron, carbon monoxide, and biliverdin, which is converted to bilirubin by the enzyme biliverdin reductase. The biliverdin and bilirubin exhibit potent antioxidant properties responsible, at least in part, for the neuroprotective effects of HO-1 [64]. NQO1 is a ubiquitous FAD-dependent flavoprotein that promotes 2-electron reductions of quinones to semiquinones thereby minimizing the generation of reactive oxygen intermediates by redox cycling and the depletion of intracellular thiols pool [65]. Hence, studies that identify possible natural sources of factors that upregulate the expression and activity of enzymes such as HO-1 and NQO1 represent a relevant contribution to the identification of dietary components with a therapeutic potential against oxidative stress characterizing several degenerative diseases.

Trx is a multifunctional and ubiquitous protein characterized by the presence of a redox-active disulfide/dithiol within its conserved active site, which functions in the reduction of protein disulfides and as a scavenger of ROS [66].* In vitro*, Trx has a protective effect against cytotoxicity mediated by ROS [67]. TrxR, a selenium-containing protein, reduces oxidized Trx. Together, Trx and TrxR operate as a powerful NADPH-dependent protein disulfide reductase system capable of repairing oxidized proteins or of maintaining levels of reduced Trx, which can directly interact with ROS. Our study demonstrates the involvement of the Trx/TrxR system in protection against the A*β*-induced cytotoxicity. In SH-SY5Y cells, exposure to A*β*_25–35_ alone determined only a weak induction of Trx and TrxR genes. Conversely, the broccoli sprouts juices quickly showed a strong inductive effect on the Trx and TrxR mRNA expression, which could account for the increased ability to counteract oxidative stress-related cell death induced by A*β*_25–35_. The involvement of Trx/TrxR in neurodegeneration has been demonstrated by Lovell and Xie [68] who showed a general reduction in the levels of Trx in various regions of human AD brains characterized by extensive oxidative damage and neuronal degeneration. Conversely, TrxR activity showed an opposite trend, pointing to a compensatory mechanism resulting from the increased oxidative stress due to Trx depletion. The same authors observed that sufficiently high levels of both components of the Trx/TrxR system, under oxidative stress conditions such as those present in AD, would ensure a neuroprotective effect. Indeed, the simultaneous exposure of primary hippocampal neurons to exogenous Trx and TrxR attenuated neuronal degeneration mediated by A*β* peptide, probably due to direct antioxidant activity of Trx. On the other hand, Venkateshappa et al. [69] observed that aging contributes to the vulnerability of those regions of the brain that are normally involved in AD. In the aging brain, the cerebral antioxidant response undergoes a significant depletion becoming increasingly inadequate to ensure the homeostasis of the redox state within neuronal cells. The age-dependent reduction of the availability of adequate levels of antioxidant enzymes, such as TrxR, and the consequent increase of oxidative stress would favor the gradual onset of neuronal dysfunction toward which a compensatory reaction that exploits the upregulation of enzymes related to cell survival (e.g., TrxR) may still not be adequately supported by a sufficient amount of critical cofactors (such as Trx).

Taken together, our findings suggest that beyond the benefits resulting from a direct action as scavengers of free radicals, antioxidant electrophilic compounds such as SFN and different classes of phenolic compounds can synergistically act as indirect stimulators of cellular endogenous antioxidant defences. By comparing two food matrices differing in polyphenols content but with a similar amount of SFN, we were able to point out consistent differences in their effectiveness in increasing intracellular levels of GSH—an essential factor for the action of antioxidant and detoxifying enzymes such as GST and GPx—and in upregulating mRNA expression or activity of other critical antioxidant enzymes such as HO-1 and NQO1 via Nrf2 activation.

## 5. Conclusions

Raw broccoli sprouts juice was shown here to protect against A*β*-induced cytotoxicity and apoptosis. This protection was exerted* via* the induction of antiapoptotic signals, such as increased Hsp70 mRNA levels, and the activation of Nrf2-ARE signalling pathway; this, through the upregulation of Nrf2-dependent antioxidant capacity, determined reduction in the A*β*-induced oxidative damages.

The results in the present study suggest that pharmacologic activation of the Nrf2 signalling pathway by broccoli sprouts juices might be a practical preventive and therapeutic strategy for AD patients. However, further studies are needed to elucidate the molecular basis of the neuroprotective effects of various chemical components of this dietary supplement and cross-talk between these and several cellular signalling cascades.

## Figures and Tables

**Table 1:** Phenolics and sulforaphane content of broccoli sprouts juices.

	Juice A	Juice B
Total polyphenols (mg GAE/mL)	1.40 ± 0.08	2.86 ± 0.33^*^
Total flavonoids (mg RE/mL)	0.44 ± 0.02	1.18 ± 0.06^***^
Total anthocyanins (*µ*g CGE/mL)	ND	29.41 ± 4.02
Sulforaphane (*µ*g/mL)	18.60 ± 1.20	20.40 ± 1.90

Mean ± SEM (*n* = 3) for total polyphenols, flavonoids, and anthocyanins.

Mean ± SEM (*n* = 5) for sulforaphane.

Student's *t*-test: ^*^ *P* < 0.05; ^***^ *P* < 0.001 versus juice A.

ND: not detectable.

**Table 2:** Antioxidant activity of broccoli sprouts juices.

Free radical scavenging activity	IC_50_ (*μ*L/mL)	
	Juice A	Juice B
NBT assay (^•^O_2_^−^)	5.17 ± 0.54	2.37 ± 0.26^*^
ABTS assay (ABTS^•+^)	2.70 ± 0.06	1.63 ± 0.03^**^
DPPH assay (DPPH^•^)	8.73 ± 0.07	2.67 ± 0.03^***^
Deoxyribose assay (^•^OH)	1.85 ± 0.03	0.77 ± 0.03^***^

Oxygen radical absorbance capacity	ORAC value (mmol/L Trolox equivalents)	
	Juice A	Juice B

ORAC assay (R-O-O^•^)	7.06 ± 0.37	11.46 ± 0.46^***^

Mean ± SEM (*n* = 3) for free radical scavenging activity.

Mean ± SEM (*n* = 5) for oxygen radical absorbance capacity.

Student's *t*-test: ^*^ *P* < 0.05; ^**^ *P* < 0.01; ^***^ *P* < 0.001 versus juice A.
